# Endoscopically Treated Third Ventricle Colloid Cysts: A Systematic Review of Surgical and Clinical Outcomes

**DOI:** 10.3390/clinpract16020029

**Published:** 2026-01-29

**Authors:** Edoardo Agosti, Sara Antonietti, Michael Viola, Marco Maria Fontanella, Alessandro Fiorindi

**Affiliations:** Neurosurgery, Department of Medical and Surgical Specialties, Radiological Sciences and Public Health, University of Brescia, 25123 Brescia, Italymarco.fontanella@unibs.it (M.M.F.); alessandro.fiorindi@asst-spedalicivili.it (A.F.)

**Keywords:** third ventricle colloid cyst, endoscopic resection, gross total resection

## Abstract

Background/Objectives: Third ventricle colloid cysts (TVCCs) are benign lesions that may cause acute hydrocephalus and, rarely, sudden death. Endoscopic resection has emerged as a minimally invasive alternative to microsurgical approaches. This systematic review aimed to evaluate the safety and efficacy of endoscopic resection of TVCCs. Methods: Following PRISMA guidelines, a systematic search of major databases was performed to identify studies reporting clinical outcomes of endoscopic resection of TVCCs. Extracted data included the surgical technique, extent of resection, complications, recurrence, and reoperations. Results: Thirty-four studies comprising 1123 patients were included. Gross total resection (GTR) was achieved in 767 patients (68.3%), with higher rates for the transforaminal (88.4%) and transeptal (86.9%) approaches (z = 0.309; *p* = 0.76). Capsule removal was performed in 87.4% and coagulation alone in 11.6%. Postoperative remnants occurred in 172 patients (17.1%). Recurrence was observed in 41 cases (3.7%) after a mean follow-up of 46.3 months, with 33 patients (2.9%) requiring reoperation. Preoperative hydrocephalus was present in 51% of cases. Septostomy and external ventricular drainage were performed in 15.7% and 15.5% of patients, respectively. Complications included memory deficits (3.6%), meningitis (3.6%), intraventricular hemorrhage (2.7%), ischemia (1.1%), shunt dependency (2.1%), and seizures (0.6%). Mortality occurred in eight patients (0.7%). Conclusions: Endoscopic management of TVCCs is associated with a low complication rate and favorable long-term outcomes. Capsule resection reduces the risk of recurrence and the need for reoperation.

## 1. Introduction

Third ventricle colloid cysts (TVCCs) are rare, benign intracranial lesions, accounting for approximately 0.5% to 1% of all brain tumors. Histologically, they are characterized by a thin collagenous wall lined with epithelium, encasing a gelatinous material of variable viscosity. These cysts are predominantly located at the anterior part of the roof of the third ventricle, often adhering to structures such as the velum interpositum or the choroid plexus near the foramen of Monro. Clinically, TVCCs present a wide spectrum of manifestations. While many remain asymptomatic and are discovered incidentally during imaging or autopsy, others can cause significant obstructive hydrocephalus, leading to increased intracranial pressure, headaches, memory disturbances, gait instability, and, in severe cases, sudden death. The risk of neurological deterioration stems from the cyst’s potential to block cerebrospinal fluid flow at the foramen of Monro, emphasizing the clinical importance of timely diagnosis and appropriate management [1,2,3].

Historically, the treatment of TVCCs has been rooted in open microsurgical techniques. The two main surgical approaches (i.e., the transcortical–transventricular route and the transcallosal approach) have been considered the gold standards for many years [4]. These techniques aim to achieve gross total resection (GTR) of the cyst, thereby eliminating the mass effect and preventing recurrence. Other methods such as ventriculoperitoneal shunting and stereotactic aspiration have also been described, particularly in patients with high surgical risks or in those requiring temporary relief from hydrocephalus. Despite the effectiveness of these traditional methods, they are associated with notable morbidity, including risks related to brain retraction, venous injury, and neurocognitive sequelae, which has driven the exploration of less invasive alternatives [5,6].

The development and refinement of neuroendoscopic techniques have profoundly influenced the management of colloid cysts. These techniques have also been successfully applied in other neurosurgical fields, including the management of intraventricular tumors and arachnoid cysts. Since their first application in the early 1980s, endoscopic approaches have gained traction as a minimally invasive alternative to open surgery. Endoscopy allows direct visualization and resection of the cyst contents and wall with reduced manipulation of surrounding brain structures. The potential advantages include shorter operative times, reduced hospital stays, lower complication rates, and faster recovery. However, concerns have been raised regarding the extent of resection (EoR) and long-term recurrence rates compared to microsurgical techniques. Nevertheless, accumulating evidence over the past two decades has increasingly supported the efficacy and safety of endoscopic procedures, positioning them as a strong contender to traditional open approaches [7].

Despite the growing adoption of endoscopic management for third ventricular colloid cysts, a comprehensive and up-to-date synthesis of the available evidence remains lacking. Most of the published literature consists of comparative studies of endoscopic versus microsurgical treatments or retrospective case series of endoscopically treated TVCCs with relatively small patient cohorts and limited follow-up durations. Furthermore, discrepancies exist in the endoscopic techniques employed, patient selection criteria, and definitions of treatment success across studies. As a result, there is a pressing need for an updated systematic review of the available data to better direct clinical decision-making and to establish the role of endoscopy in TVCCs treatment [8].

The objective of the present systematic review is to comprehensively analyze the existing literature on the endoscopic treatment of TVCCs, with the aim of clarify the efficacy, safety profile, recurrence rates, and long-term outcomes associated with endoscopic techniques.

## 2. Materials and Methods

### 2.1. Literature Search Strategy

This systematic review was conducted in accordance with the Preferred Reporting Items for Systematic Reviews and Meta-Analyses (PRISMA) guidelines [9]. A comprehensive search was performed across three electronic databases: PubMed, Scopus, and Web of Science. The search covered the period from January 1983 to January 2025, employing the following strategic search string: “((Colloid cyst AND third ventricle) AND (endoscopic treatment) NOT (microsurgical))”. Initially, titles were screened to identify studies exclusively reporting on endoscopic management. Subsequently, abstracts were reviewed, and full texts of potentially eligible studies were retrieved and assessed for inclusion.

Studies were selected according to predefined inclusion criteria: (1) publications written in English; (2) availability of anamnestic and clinical data including the number of patients, the mean age, cyst size, and presence of hydrocephalus; (3) surgical data reporting the type of endoscope used, the surgical approach, and the surgical technique; (4) postoperative follow-up data including morbidity, recurrence rates, and any reoperations; and (5) studies comprising a minimum of five patients.

Exclusion criteria included (1) editorials, case reports, literature reviews, and meta-analyses; (2) studies lacking clear methodological descriptions or results; and (3) articles not written in English.

All identified records were imported into the Zotero (v. 7.0) reference management software, and duplicates were systematically removed. This systematic review was not registered.

### 2.2. Data Extraction

Data were extracted independently from the selected articles using a standardized form. Extracted variables included: demographic and clinical characteristics (number of patients, mean age, cyst size, and incidence of preoperative hydrocephalus), surgical details (type of endoscope employed, surgical route, type of endoscopic intervention), and surgical outcomes. Study screening and data extraction were performed independently by two reviewers. Any discrepancies were resolved through discussion and, when necessary, by involving a third reviewer.

The surgical approach was categorized into three groups: (1) cyst evacuation (CE); (2) capsule coagulation (CC), including a preliminary CE; and (3) capsule resection (CR), including preliminary CE and CC. Gross total resection was defined as absence of visible TVCC remnants at postoperative computer tomography (CT) scan.

Postoperative data collected included intraoperative and postoperative complications, mortality, regrowth rates (i.e., TVCC remnant growth or TVCC recurrence), and the second-surgery rate.

### 2.3. Statistical Analysis

This study was designed as a descriptive systematic review; therefore, no formal meta-analysis or inferential statistical testing was performed. Extracted data from the included studies were summarized narratively and using descriptive statistics. Categorical variables (e.g., surgical approach, type of capsule management, presence of hydrocephalus, complications, recurrence, and reoperation rates) were reported as absolute numbers and percentages. Continuous variables (e.g., age, cyst size, and follow-up duration) were summarized as means or medians when available.

No heterogeneity assessment (I^2^ statistic), subgroup analysis, sensitivity analysis, or publication bias evaluation was conducted due to the methodological heterogeneity of the included retrospective case series and the absence of patient-level data.

### 2.4. Risk of Bias Assessment

**Figure 1:**
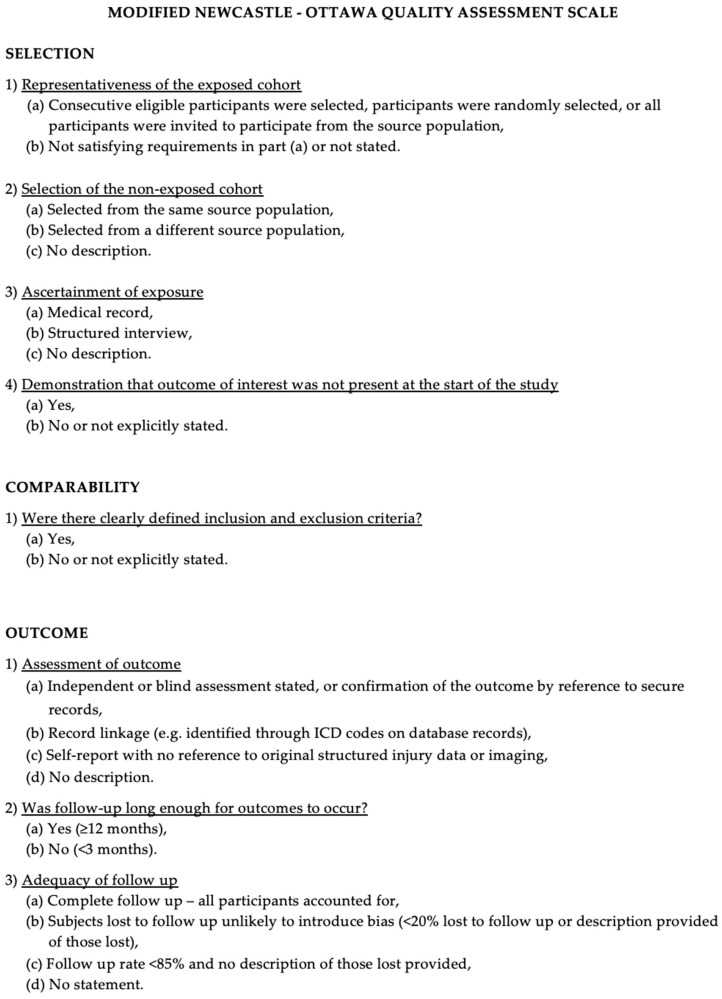
The Modified NOS.

The methodological quality and risk of bias of the included studies were assessed using the Newcastle–Ottawa Scale (NOS) [10]. This tool evaluates three domains: selection of study groups, comparability between groups, and ascertainment of outcomes. The maximum achievable score was 9, with studies scoring ≥7 considered high quality. Two authors independently performed quality assessments, and disagreements were resolved by consensus with a third reviewer (Figure 1 and Table S1).

## 3. Results

### 3.1. PRISMA

**Figure 2:**
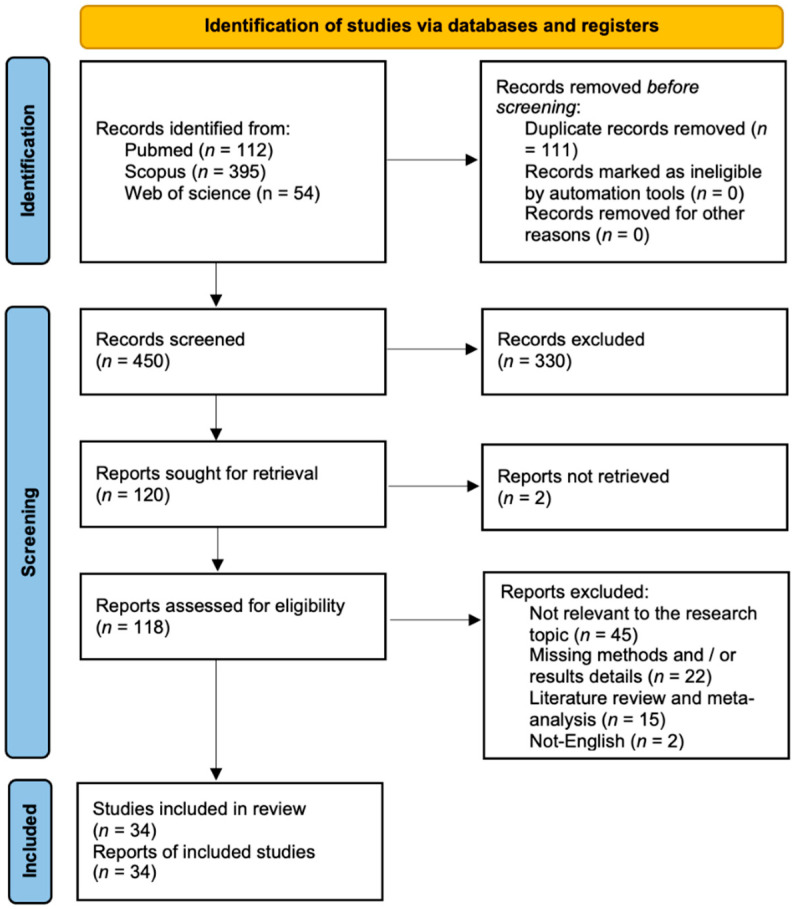
Prisma flow chart.

A total of 450 records were identified following the removal of duplicates. After the initial screening of titles and abstracts, 120 studies were deemed appropriate for full-text evaluation. Upon detailed review, 118 articles satisfied the eligibility criteria. Ultimately, 34 studies were included in the final systematic review. The remaining 84 articles were excluded for the following reasons: (1) lack of relevance to the research topic (45 articles); (2) insufficient methodological description and/or absence of clearly reported results (22 articles); (3) literature reviews or meta-analyses (15 articles); (4) publications in a language other than English (2 articles). The study selection process is summarized in Figure 2. The PRISMA 2020 checklist is reported in Supplementary Material (File S1).

### 3.2. Clinical and Surgical Data

A summary of the main demographic and surgical characteristics of the included studies is presented in Table 1.

This systematic review encompasses studies published between 1998 and 2023, collectively analyzing a cohort of 1123 patients who underwent endoscopic management for TVCCs. The median patient age was 38.9 years, and the median cyst size was 13.9 mm. A significant proportion of patients, specifically 573 individuals (51%), presented with preoperative hydrocephalus. Concerning the endoscopic instrumentation, two types of endoscopes were employed: rigid and flexible. Rigid endoscopes were utilized in most cases (1078 patients, 96%), while flexible endoscopes were used in 45 cases (4%). Three distinct surgical approaches for cyst resection were identified: transforaminal, transeptal, and interforniceal. The transforaminal route was the most employed, accounting for 94.7% of cases, whereas the transeptal and interforniceal approaches were utilized in 6.3% and 0.1% of cases, respectively.

Additional intraoperative procedures performed during endoscopic management were also analyzed. Septostomy was required in 173 patients (15.7%), external ventricular drainage (EVD) was placed in 171 patients (15.5%), and a third ventriculostomy was performed in 23 cases (2.1%).

Finally, the types of cyst removal techniques utilized were reviewed. Notably, no cases involved simple evacuation of cyst contents alone. Capsule coagulation was performed in 130 patients (11.6%). The predominant technique, employed in 982 cases (87.4%). A detailed summary of these findings is provided in Table S2.

### 3.3. Surgical and Clinical Outcomes

The mortality rate was 0.7% (8 patients). Neurological complications constituted a significant postoperative concern. Shunt dependency was documented in 24 patients (2.1%). Persistent memory deficits, predominantly secondary to intraoperative injury to limbic structures, were reported in 40 patients (3.6%). Postoperative meningitis occurred in an equivalent proportion of cases. Seizures were documented in 7 patients (0.6%). Vascular complications were also recorded: ischemic events occurred in 12 patients (1.1%), while intraventricular hemorrhage emerged as the most prevalent vascular complication, affecting 30 patients (2.7%). Intra- or extra-axial hemorrhages were noted in 8 patients (0.7%). Finally, GTR was achieved in 767 patients (68.3%) (Table S3). The GTR rate was 88.4% for the transforaminal route and 86.9% for the transeptal route. A detailed synthesis of these findings is presented in Table S4.

After postoperative CT scan, TVCC remnants were identified in 172 patients (17.1%). The mean follow-up duration was 46.3 months. During this extended surveillance period, TVCC regrowth was detected in 41 patients (3.7%). Among those with documented recurrence, 33 patients (2.9%) presented a symptomatic regrowth and ultimately required a second surgery. A detailed overview and a summary of these results are presented in Tables S5 and S6, respectively.

## 4. Discussion

Endoscopic resection has increasingly been proposed as a minimally invasive alternative to traditional microsurgical routes for the treatment of TVCCs, offering lower morbidity while achieving satisfactory resection rates. Within this evolving landscape, our findings provide an updated synthesis of outcomes associated with endoscopic techniques. According to our results, most patients presented with hydrocephalus and underwent surgery using rigid endoscopes, primarily via a transforaminal route. Beyond preoperative hydrocephalus, the most frequently reported indications for surgery included progressive tumor growth, symptoms of raised intracranial pressure (headache, nausea, and vomiting), new-onset seizures, and focal neurological deficits. Capsule resection was the predominant technique, while simple evacuation was rarely employed. Gross total resection was achieved in most cases, while recurrence and the need for reoperation were infrequent, supporting the increasing adoption of endoscopy as an effective treatment option for TVCCs.

The demographic and clinical characteristics of patients in our review are consistent with those reported in previous studies. Greenlee et al. [15], Wilson et al. [22], and Roth et al. [38], for instance, described similar age distributions and a predominance of male patients, with reported mean patient ages in the 30 s to 40 s. The high rate of hydrocephalus observed in our series aligns with findings from Sribnick et al. [25], and Boogaarts et al. [19], underscoring the typical clinical presentation resulting from obstruction at the foramen of Monro. Several authors, including Chibbaro et al. [20], Ibáñez-Botella et al. [24], and Zymberg et al. [35], have highlighted hydrocephalus as a key determinant of symptom onset and a crucial factor in guiding the timing of surgical intervention.

According to our results, studies by Pinto et al. [17], El-Ghandour et al. [18], and Samadian et al. [30] also reported a strong preference for CR whenever technically feasible. While CC and partial resections may provide short-term symptom relief, several authors, such as Hoffman et al. [21] and Beaumont et al. [37], have cautioned against leaving cyst wall remnants due to the associated risk of recurrence. Notably, our observed recurrence rate of 3.7% is lower than that reported in earlier studies, including Longatti et al. [13] and Birski et al. [29], which may reflect improvements in endoscopic technology and growing surgical expertise. Moreover, some of the most recent studies in our review (Lin et al. [33], Beaumont et al. [37], and Peron et al. [39]) reflect a trend toward higher GTR rates with endoscopic techniques, potentially owing to advances such as dual-instrument and dual-port approaches. These findings suggest a narrowing gap between endoscopy and microsurgery in terms of oncological efficacy, albeit longer follow-up data are still needed to confirm durability.

Gross total resection was defined as the absence of surgical remnants on immediate postoperative imaging. However, as emphasized by Hoffman et al. [21] and others, intraoperative visualization may be a more accurate determinant, as small remnants attached to critical structures may not be apparent on postoperative scans. Our strict imaging-based definition may therefore underestimate subtle residuals, contributing to recurrence underreporting. In our review, the GTR rate was 68.3%, with most failures attributed to anatomical constraints or adhesions to vital structures. This is notably lower than the GTR rates reported in microsurgical series, which often exceed 90% [21,35]. As noted by Decq et al. [12] and Grondin et al. [14], limitations in bimanual dissection and constrained visualization remain inherent challenges in endoscopic surgery. Nonetheless, several recent studies, including Lin et al. [33] and Peron et al. [39], have reported endoscopic GTR rates approaching those of microsurgery, particularly with the adoption of advanced dual-instrument techniques. Even if microsurgical approaches achieve higher GTR and lower recurrence rates (Samadian et al. [30]; Zymberg et al. [35]), the trade-off lies in the higher morbidity associated with open surgery, including longer hospital stays, increased risk of venous infarction, and cognitive deficits (Boogaarts et al. [19]; Roth et al. [38]). So, while endoscopy may not always match the radicality of microsurgery, it offers a safe and effective treatment with lower complication rates, especially when performed by experienced hands (e.g., Cappabianca et al. [41]; de Divitiis et al. [42]).

Although some authors such as Hellwig et al. [16] and Iacoangeli et al. [23] reported higher rates of intraoperative complications with endoscopic procedures, our complication rates remain relatively low. For instance, postoperative memory deficits (3.6%) and shunt dependency (2.1%) are consistent with or lower than those in series by Boogarts et al. [19], Azab et al. [31], and Stachura et al. [34]. Notably, intraventricular hemorrhage, the most common vascular complication in our dataset (2.7%), was also reported by Brunori et al. [7] and Unal et al. [40] at similar frequencies. The low seizure rate observed (0.6%) further underscores the minimally invasive nature of endoscopic approaches when compared to transcortical microsurgical routes, which have been more frequently associated with postoperative seizures [38].

We reported a regrowth rate of 3.7%, comparable to that observed by Hoffman et al. [21] and Vorbau et al. [32], both of whom noted that recurrence is predominantly seen in cases where the capsule is not completely excised. Interestingly, several studies in our review, including Sribnick et al. [25], Stachura et al. [34], and Guive Sharifi et al. [27], reported no recurrences despite partial resections, underscoring the complexity of correlating cyst remnants with recurrence risk. This remains a debated topic in the literature. Some authors, such as Decq et al. [12] and Longatti et al. [13], argue that coagulated remnants may remain stable, while others, like Beaumont et al. [37] and Roth et al. [38], suggest that even small residuals can regrow, especially in younger patients with longer life expectancy [36,43,44,45,46,47,48,49,50,51,52,53,54].

### Limitations of the Study

The included studies are mostly retrospective and heterogeneous in methodology, which introduces selection bias and limits the strength of pooled conclusions. The lack of standardized definitions for GTR, inconsistent use of intraoperative imaging, and variable follow-up durations further complicate data interpretation, and the short follow-up reported in most studies may lead to an underestimation of late recurrences Most studies had limited sample sizes, and only a few included long-term follow-ups exceeding five years, which is critical when evaluating recurrence. Additionally, while we excluded mixed modality studies to focus on endoscopy, this precludes direct comparative analysis with microsurgical techniques. In several studies, GTR was defined using only postoperative CT, which may fail to detect small capsule remnants and could lead to underestimation of residual tumor and recurrence rates. Finally, although our review includes a large patient cohort, publication bias cannot be excluded, as positive outcomes are more likely to be reported in the literature.

## 5. Conclusions

Endoscopic management of TVCCs is associated with a low complication rate and favorable long-term outcomes. Capsule resection, when feasible, is the preferred technique, as it significantly reduces the risk of recurrence and the need for reoperation.

## Figures and Tables

**Table 1:** Demographic and surgical characteristics from individual studies included in the systematic review. CR = capsule resection; CC = capsule coagulation; EVD = External Ventricular Drain; N/A = not applicable; TV = third ventriculostomy.

Author, Years	Anamnestic Data				Surgical Data			
	Patients N	Age Mean— Years	Cyst Size Mean—mm	Preoperative Hydrocephalus N (%)	Type of Endoscope	Surgical Route N (%)	Other Surgical Procedures N (%)	Type of Surgery N (%)
Abdou et al., 1998 [11]	13	39.0	NA	13 (100)	Rigid	Transforaminal 13 (100)	/	CR 13 (100)
Decq et al., 1998 [12]	19	NA	23.0	2 (10.5)	Rigid	Transforaminal 19 (100)	NA	CR 19 (100)
Longatti et al., 2006 [13]	61	41	32	53 (86.9)	Rigid	Transforaminal 61 (100)	/	CC 32 (52.4); CR 6 (9.8)
Grondin et al., 2007 [14]	25	45.1	13.5	22 (88)	Flexible	Transeptal 25 (100)	/	CC 22 (88); CR 3 (12)
Greenlee et al., 2008 [15]	29	32.4	18	NA	Rigid	Transforaminal 29 (100)	Septostomy 1 (3.4)	CR 29 (100)
Hellwig et al., 2008 [16]	20	43	NA	20 (100)	Flexible	Transforaminal 20 (100)	EVD 20 (100)	CR 20 (100)
Pinto et al. 2009 [17]	11	38	14.4	11 (100)	Rigid	Transforaminal 11 (100)	/	CC 11 (100)
El-Ghandour 2009 [18]	10	42.5	NA	10 (100)	Rigid	Transforaminal 10 (100)	TV 2 (20); septostomy 2 (20)	CC 10 (100)
Mishra et al., 2010 [6]	59	32.0	NA	59 (100)	Rigid	Transforaminal 59 (100)	Septostomy 6 (10.2); EVD 14 (23.7)	CR 59 (100)
Delitala et al., 2011 [5]	7	51.8	12.8	4 (57.1)	Rigid	Transforaminal 7 (100)	/	CR 7 (100)
Boogaarts et al., 2011 [19]	91	43	16.4	16 (17.6)	Rigid	Transforaminal 91 (100)	TV 7 (7.7); septostomy 7 (7.7)	CR 91 (100)
Chibbaro et al., 2013 [20]	29	34	2.1	25 (86.2)	Rigid	Transforaminal 29 (100)	Septostomy 25 (86.2); EVD 2 (6.9)	CR 29 (100)
Hoffman et al., 2013 [21]	56	41	1.3	37 (66)	Rigid	Transforaminal 56 (100)	EVD 24 (42.9)	CC 9 (16.1); CR 45 (80.4)
Wilson et al., 2013 [22]	22	47	1.2	19 (86.3)	Rigid	Transforaminal 22 (100)	EVD 17 (77.2)	CC 1 (4.5); CR 21 (95.5)
Iacoangeli et al., 2014 [23]	5	48.6	26.4	5 (100)	Rigid	NA	TV 2 (40); septostomy 4 (80)	CR 5 (100)
Ibáñez-Botella et al., 2014 [24]	24	39.6	16.3	2 (8.30)	Rigid	Transforaminal 24 (100)	TV 2 (8.3); septostomy 11 (45.8)	CR 24 (100)
Sribnick et al., 2014 [25]	56	NA	9.80	33 (60.0)	Rigid	Transforaminal 56 (100)	Septostomy 43 (76.8) T-v 2 (3.60)	CR 56 (100)
Raouf et al., 2015 [26]	90	40.3	NA	NA	Rigid	Transforaminal 90 (100)	/	CR 79 (87.8)
Guive Sharifi et al., 2015 [27]	18	35.3	26.0	0 (0)	Rigid	Transforaminal 18 (100)	EVD 6 (33.3)	CR 18 (100)
Stachura et al., 2009 [28]	5	43.0	NA	0 (0)	Rigid	Transforaminal 5 (100)	/	CR 5 (100)
Birski et al., 2016 [29]	27	37.2	12.5	23 (85.1)	Rigid	Transforaminal 27 (100)	/	CR 27 (100)
Samadian et al., 2018 [30]	112	36.2	NA	NA	Rigid	Transforaminal 112 (100)	EVD 68 (60.7)	CR 112 (100)
Brunori et al., 2018 [7]	22	46.0	1.50	19 (86.4)	Rigid	Transforaminal 13 (59); transeptal 9 (41)	EVD 1 (4.50)	CR 22 (100)
Azab et al., 2019 [31]	19	25.0	12.7	18 (94.7)	Rigid	Transforaminal 19 (100)	/	CR 19 (100)
Vorbau et al., 2019 [32]	20	39.0	NA	19 (95.0)	Rigid	Transforaminal 20 (100)	/	CR 20 (100)
Lin et al., 2020 [33]	16	50.0	14	12 (75.0)	Rigid	Transforaminal 16 (100)	EVD 3 (18.8)	CR 16 (100)
Stachura et al., 2021 [34]	58	NA	NA	NA	Rigid	Transforaminal 58 (100)	EVD 5 (8.60)	CR 58 (100)
Zymberg et al., 2021 [35]	45	35.4	26.8	45 (100)	Rigid	Transforaminal 32 (71.1); transeptal 11 (24.5); interforniceal 2 (4.4)	/	CC 45 (100)
Ali Alkhaibary et al., 2021 [36]	21	37.7	16.2	NA	Rigid	Transforaminal 21 (100)	EVD 1 (4)	CR 21 (100)
Arnaout et al., 2021 [8]	12	28.5	22.0	12 (100)	Rigid	NA	/	CR 12 (100)
Beaumont et al., 2022 [37]	33	40.2	11.9	16 (48.5)	Rigid	Transforaminal 33 (100)	Septostomy 29 (87.9) EVD 9 (27.2)	CR 33 (100)
Roth et al., 2022 [38]	62	12.8	12.6	61 (98.4)	Rigid	Transforaminal 62 (100)	Septostomy 45 (87); TV 8 (12.9)	CR 62 (100)
Peron et al., 2023 [39]	5	45	12	4 (80)	Rigid	Transforaminal 5 (100)	/	CR 5 (100)
Unal et al., 2023 [40]	21	41	13.9	13 (62)	Rigid	Transforaminal 21 (100)	EVD 1 (4.8)	CR 21 (100)

## Data Availability

Data available in a publicly accessible repository.

## Supplementary Materials

The following supporting information can be downloaded at https://www.mdpi.com/article/10.3390/clinpract16020029/s1. File S1: PRISMA 2020 checklist; Table S1: Summary of pooled demographic and surgical data of all included patients; Table S2: Postoperative complications and GTR reported in individual studies; Table S3: Overall incidence of postoperative complications and gross total resection across all studies; Table S4: Follow-up data reported in individual studies included in this review; Table S5: Summary of pooled follow-up outcomes across all included studies; Table S6: Summary of the Newcastle–Ottawa Scale (NOS) scores for each included study. References [5,6,7,8,11,12,13,14,15,16,17,18,19,20,21,22,23,24,25,26,27,28,29,30,31,32,33,34,35,36,37,38,39,40] are cited in the supplementary materials.

## Abbreviations

The following abbreviations are used in this manuscript:

TVCCs	Third ventricle colloid cysts
GTR	Gross total resection
EVD	external ventricular drainage
CR	Capsule resection
CC	Capsule coagulation
CE	Cyst evacuation
