# Supplementary material for: Endoscopically Treated Third Ventricle Colloid Cysts: A Systematic Review of Surgical and Clinical Outcomes
Source: Clin Pract. 2026 Jan 29;16(2):29. doi: 10.3390/clinpract16020029 (PMC12939540; doi:10.3390/clinpract16020029)
Supplement: Supplementary file 1 [file clinpract-16-00029-s001.zip › Table.pdf]

Table S1. Summary of pooled demographic and surgical data of all included patients.

Anamnestic data				Surgical data										
Patients N	Median age (year)	Median cyst size (mm)	Preoperative hydrocephalus N (%)	Type of endoscope N (%)		Surgical route N (%)			Other surgical procedures N (%)			Type of surgery N (%)		
				Rigid	Flexible	Transforaminal	Transeptal	Interforniceal	Septostomy	TV	EVD	CE	CC	CR
1123	38.9	13.9	573 (51)	1078 (96)	45 (4)	1051 (95.6)	46 (4.2)	2 (0.2)	173 (15.7)	23 (2.1)	171(15.5)	0	130 (11.6)	982 (87.4)

Table S2: Postoperative complications and GTR reported in individual studies.

Author, years	Surgical complications								GTR N (%)
	**Mortality N (%)**	**Shunt dependency N (%)**	**Permanent memory deficit N (%)**	**Meningitis N (%)**	**Seizures N (%)**	**Ischemia N (%)**	**Intraventricular haemorrhage N (%)**	**Extra or intra axial hematoma N (%)**	
Abdou et al., 1998 [1]	0	0	1 (7.7)	0	0	0	0	0	0
Decq et al., 1998 [2]	0	0	0	0	0	0	0	0	12 (63.2)
Longatti et al., 2006 [3]	0	0	0	1 (1.6)	0	1	3 (4.9)	1 (1.6)	6 (9.8)
Grondin et al., 2007 [4]	0	0	0	0	1 (4)	1 (4)	0	0	3 (12)
Greenlee et al., 2008 [5]	0	0	0	2 (6.9)	0	0	0	0	29 (100)
Hellwig et al., 2008 [6]	0	1 (5)	1 (5)	1 (5)	0	1 (5)	1 (5)	0	6 (30)
Pinto et al. 2009 [7]	0	0	0	1 (9.1)	0	0	0	0	0
El-Ghandour 2009 [8]	0	0	0	0	0	0	0	0	0
Mishra et al., 2010 [9]	0	0	0	0	0	0	14 (23.7)	0	46 (78)
Delitala et al., 2011 [10]	0	0	0	0	0	0	0	0	0
Boogaarts et al., 2011 [11]	1 (1.1)	3 (3.3)	1 (1.1)	10 (11)	1 (1.1)	1 (1.1)	0	0	41(45.1)
Chibbaro et al., 2013 [12]	0	0	0	1 (3.5)	0	0	1 (3.5)	0	25 (86.2)
Hoffman et al., 2013 [13]	0	0	0	1 (1.8)	0	0	1 (1.8)	0	45 (80.4)
Wilson et al., 2013 [14]	0	0	0	0	0	0	0	1 (4.6)	21 (95.5)
Iacoangeli et al., 2014 [15]	0	0	0	0	0	0	0	0	5 (100)
Ibáñez-Botella et al., 2014 [16]	0	0	2 (8.3)	1 (4.2)	0	0	3	0	23 (95.8)
Sribnick et al., 2014 [17]	2 (3.6)	0	6 (10.7)	3 (5.4)	0	0	0	1 (1.8)	56 (100)
Raouf et al., 2015 [18]	0	0	0	5 (5.6)	0	2 (2.2)	0	0	79 (87.8)
Guive Sharifi et al., 2015 [19]	1 (5.6)	0	0	0	0	0	0	0	16 (88.9)

Stachura et al., 2009 [20]	0	0	2 (40)	0	0	0	0	0	5 (100)
Birski et al., 2016 [21]	0	0	0	0	0	0	1 (3.7)	0	27 (100)
Samadian et al., 2018 [22]	2 (1.8)	4 (3.6)	5 (4.5)	9 (8)	0	4 (3.6)	0	0	103 (92)
Brunori et al., 2018 [23]	0	0	1 (4.6)	0	0	0	1 (4.6)	1 (4.6)	16 (72.7)
Azab et al., 2019 [24]	0	0	0	0	0	0	1 (5.3)	0	17 (77.3)
Vorbau et al., 2019 [25]	0	0	0	1 (5)	0	0	0	2 (10)	19 (95)
Lin et al., 2020 [26]	0	0	0	0	0	0	0	0	16 (100)
Stachura et al., 2021 [27]	1 (1.7)	5 (8.6)	4 (6.9)	0	4 (6.9)	0	0	0	47 (81)
Zymberg et al., 2021 [28]	0	0	0	1 (2.2)	0	0	2 (4.4)	0	3 (6.7)
Ali Alkhaibary et al., 2021 [29]	0	0	0	1 (4.8)	0	0	0	1 (4.8)	21 (46.7)
Arnaout et al., 2021 [30]	0	0	2 (16.7)	0	0	0	0	0	5 (41.7)
Beaumont et al., 2022 [31]	0	0	2 (6.1)	0	0	0	1 (3)	0	12 (36.4)
Roth et al., 2022 [32]	1 (1.6)	7 (11.3)	12 (19.4)	1 (1.6)	1 (1.6)	1 (1.6)	0	1 (1.6)	41 (66.1)
Peron et al., 2023 [33]	0	0	0	0	0	0	0	0	5 (100)
Unal et al., 2023 [34]	0	1 (4.8)	1 (4.8)	1 (4.8)	0	1 (4.8)	1 (4.8)	0	17 (81)

Table S3: Overall incidence of postoperative complications and gross total resection across all studies.

**Surgical complications N (%)**								**GTR N (%)**
**Mortality**	**Shunt dependency**	**Permanent memory deficits**	**Meningitis**	**Seizures**	**Ischemia**	**Intraventricular haemorrhage**	**Extra or intra axial haematoma**	
8 (0.7)	24 (2.1)	40 (3.6)	40 (3.6)	7 (0.6)	12 (1.1)	30 (2.7)	8 (0.7)	767 (68.3)

Table S4: Follow-up data reported in individual studies included in this review.

Author, years	Follow up data		
	Follow up mean (months)	Regrowth N (%)	Second surgery N (%)
Abdou et al.,1998 [1]	48	0	0
Decq et al., 1998 [2]	15.3	1 (5.3)	0
Longatti et al., 2006 [3]	32	7 (11.5)	0
Grondin et al., 2007 [4]	38.5	1 (4)	0
Greenlee et al., 2008 [5]	88	1 (3.5)	0
Hellwig et al., 2008 [6]	64.1	0	0
Pinto et al. 2009 [7]	33	0	0
El-Ghandour 2009 [8]	24	0	0
Mishra et al., 2010 [9]	13	0	0
Delitala et al., 2011 [10]	24	0	0
Boogaarts et al., 2011 [11]	39	12 (13.2)	6
Chibbaro et al., 2013 [12]	34	0	0
Hoffman et al., 2013 [13]	40.4	1 (1.8)	2 (3.6)
Wilson et al., 2013 [14]	19.8	0	0
Iacoangeli et al., 2014 [15]	68.4	0	0
Ibáñez-Botella et al., 2014 [16]	54	0	0
Sribnick et al., 2014 [17]	14.9	1 (1.8)	0
Raouf et al., 2015 [18]	62	0	0
Guive Sharifi et al., 2015 [19]	44.4	0	0
Stachura et al., 2009 [20]	24.0	0	0
Birski et al., 2016 [21]	43.5	2 (7.4)	2 (7.4)
Samadian et al., 2018 [22]	NA	4	0
Brunori et al., 2018 [23]	62	0	0
Azab et al., 2019 [24]	27.7	0	1 (5.3)
Vorbau et al., 2019 [25]	188	0	0
Lin et al., 2020 [26]	10	0	0
Stachura et al., 2021 [27]	18.0	3 (5.17)	0
Zymberg et al., 2021 [28]	NA	0	2 (4.45)
Ali Alkhaibary et al., 2021 [29]	85.4	0	0
Arnaout et al., 2021 [30]	59	1 (8.34)	0
Beaumont et al., 2022 [31]	12	7 (21.2)	3 (9.1)
Roth et al., 2022 [32]	49.5	0	17 (27.4)
Peron et al., 2023 [33]	3	0	0
Unal et al., 2023 [34]	14	0	0

Table S5: Summary of pooled follow-up outcomes across all included studies.

Follow-up data		Second surgery N (%)
Median follow-up (months)	Regrowth N (%)	
46.3	41 (3.7)	33 (2.9)

Table S6: Summary of the Newcastle–Ottawa Scale (NOS) scores for each included study.

Study	Selection	Comparability	outcome	Total
Abdou et al.,1998 [1]	3	1	3	7
Ali Alkhaibary et al., 2021 [29]	4	1	3	8
Arnaout et al., 2021 [30]	4	1	3	8
Azab et al., 2019 [24]	3	1	3	7
Beaumont et al., 2022 [31]	4	2	3	9
Birski et al., 2016 [21]	3	1	3	7
Boogaarts et al., 2011 [11]	3	1	3	7
Brunori et al., 2018 [23]	3	1	3	7
Chibbaro et al., 2013 [12]	3	1	3	7
Decq et al., 1998 [2]	3	1	3	7
Delitala et al., 2011 [10]	3	1	3	7
El-Ghandour 2009 [8]	3	1	3	7
Greenlee et al., 2008 [5]	3	1	3	7
Grondin et al., 2007 [4]	3	1	3	7
Guive Sharifi et al., 2015 [19]	3	1	3	7
Hellwig et al., 2008 [6]	3	1	3	7
Hoffman et al., 2013 [13]	3	1	3	7
Iacoangeli et al., 2014 [15]	3	1	3	7
Ibáñez-Botella et al., 2014 [16]	3	1	3	7
Lin et al., 2020 [26]	4	1	3	8
Longatti et al., 2006 [3]	3	1	3	7
Mishra et al., 2010 [9]	3	1	3	7
Peron et al., 2023 [33]	3	1	3	7
Pinto et al. 2009 [7]	3	1	3	7
Raouf et al., 2015 [18]	3	1	3	7
Roth et al., 2022 [32]	4	1	3	8
Samadian et al., 2018 [22]	3	1	3	7
Sribnick et al., 2014 [17]	3	1	3	7
Stachura et al., 2009 [20]	3	1	3	7
Stachura et al., 2021 [27]	4	0	3	7
Unal et al., 2023 [34]	4	0	3	7
Vorbau et al., 2019 [25]	3	1	3	7
Wilson et al., 2013 [14]	3	1	3	7
Zymberg et al., 2021 [28]	3	1	3	7

Pubmed

(("colloid cyst"[Title/Abstract] OR "colloid cysts"[Title/Abstract]) AND ("third ventricle"[Title/Abstract] OR "third ventricular"[Title/Abstract]) AND (endoscop* OR "endoscopic resection" OR "neuroendoscopy") AND (outcome* OR complication* OR "gross total resection" OR recurrence)) NOT (microsurg* OR open surgery)

Scopus

(TITLE-ABS-KEY("colloid cyst" OR "colloid cysts") AND TITLE-ABS-KEY("third ventricle" OR "third ventricular") AND TITLE-ABS-KEY(endoscop* OR "endoscopic treatment" OR neuroendoscop*) AND TITLE-ABS-KEY(outcome* OR complication* OR "gross total resection" OR recurrence)) AND NOT TITLE-ABS-KEY(microsurg*)

WEB of SCIENCE

TS = (("colloid cyst" OR "colloid cysts") AND ("third ventricle" OR "third ventricular") AND (endoscop* OR "endoscopic resection" OR neuroendoscop*) AND (outcome* OR complication* OR "gross total resection" OR recurrence)) NOT TS = (microsurg*)

References

1. Abdou, M.S.; Cohen, A.R. Endoscopic treatment of colloid cysts of the third ventricle. Technical note and review of the liter-ature. *J Neurosurg.* **1998**, *89(6)*, 1062–8. https://doi.org/10.3171/jns.1998.89.6.1062.
2 Decq, P.; Le Guerinel, C.; Brugières, P.; Djindjian, M.; Silva, D.; Kéravel, Y.; Melon, E.; Nguyen, J.P. Endoscopic management of colloid cysts. *Neurosurgery* **1998**, *42*, 1288–1294; discussion 1294–1296.
3. Longatti, P.; Godano, U.; Gangemi, M.; Delitala, A.; Morace, E.; Genitori, L.; Alafaci, C.; Benvenuti, L.; Brunori, A.; Cereda, C.; et al. Cooperative study by the Italian neuroendoscopy group on the treatment of 61 colloid cysts. *Childs Nerv. Syst.* **2006**, *22*, 1263–1267.
4. Grondin, R.T.; Hader, W.; MacRae, M.E.; Hamilton, M.G. Endoscopic versus microsurgical resection of third ventricle colloid cysts. *Can. J. Neurol. Sci.* **2007**, *34*, 197–207.
5. Greenlee, J.D.W.; Teo, C.; Ghahreman, A.; Kwok, B. Purely endoscopic resection of colloid cysts. *Neurosurgery* **2008**, *62*, 51–55; discussion 55–56.
6. Hellwig, D.; Bauer, B.L.; Schulte, M.; Gatscher, S.; Riegel, T.; Bertalanffy, H. Neuroendoscopic treatment for colloid cysts of the third ventricle: The experience of a decade. *Neurosurgery* **2008**, *62*, 1101–1109.
7. Pinto, F.C.G.; Chavantes, M.C.; Fonoff, E.T.; Teixeira, M.J. Treatment of colloid cysts of the third ventricle through neuroendoscopic Nd: YAG laser stereotaxis. *Arq. Neuropsiquiatr.* **2009**, *67*, 1082–1087.
8. El-Ghandour, N.M.F. Endoscopic treatment of third ventricular colloid cysts: A review including ten personal cases. *Neurosurg. Rev.* **2009**, *32*, 395–402.
9. Mishra, S.; Chandra, P.S.; Suri, A.; Rajender, K.; Sharma, B.S.; Mahapatra, A.K. Endoscopic management of third ventricular colloid cysts: Eight years' institutional experience and description of a new technique. *Neurol. India* **2010**, *58*, 412–417.
10. Delitala, A.; Brunori, A.; Russo, N. Supraorbital endoscopic approach to colloid cysts. *Neurosurgery* **2011**, *69*, ons176–ons182; discussion ons182–ons183.
11. Boogaarts, H.D.; Decq, P.; Grotenhuis, J.A.; Le Guérinel, C.; Nseir, R.; Jarraya, B.; Djindjian, M.; Beems, T. Long-term results of the neuroendoscopic management of colloid cysts of the third ventricle: A series of 90 cases. *Neurosurgery* **2011**, *68*, 179–187.
12. Chibbaro, S.; Champeaux, C.; Poczos, P.; Cardarelli, M.; Di Rocco, F.; Iaccarino, C.; Servadei, F.; Tigan, L.; Chaussemy, D.; George, B.; et al. Anterior trans-frontal endoscopic management of colloid cyst: An effective, safe, and elegant way of treatment. Case series and technical note from a multicenter prospective study. *Neurosurg. Rev.* **2014**, *37*, 235–241; discussion 241.

13. Hoffman, C.E.; Savage, N.J.; Souweidane, M.M. The significance of cyst remnants after endoscopic colloid cyst resection: A retrospective clinical case series. *Neurosurgery* **2013**, *73*, 233–237; discussion 237–239.
14. Wilson, D.A.; Fusco, D.J.; Wait, S.D.; Nakaji, P. Endoscopic resection of colloid cysts: Use of a dual-instrument technique and an anterolateral approach. *World Neurosurg.* **2013**, *80*, 576–583.
15. Iacoangeli, M.; di Somma, L.G.M.; Di Rienzo, A.; Alvaro, L.; Nasi, D.; Scerrati, M. Combined endoscopic transforaminal-transchoroidal approach for the treatment of third ventricle colloid cysts. *J. Neurosurg.* **2014**, *120*, 1471–1476.
16. Ibáñez-Botella, G.; Domínguez, M.; Ros, B.; De Miguel, L.; Márquez, B.; Arráez, M.A. Endoscopic transchoroidal and transforaminal approaches for resection of third ventricular colloid cysts. *Neurosurg. Rev.* **2014**, *37*, 227–234; discussion 234.
17. Sribnick, E.A.; Dadashev, V.Y.; Miller, B.A.; Hawkins, S.; Hadjipanayis, C.G. Neuroendoscopic colloid cyst resection: A case cohort with follow-up and patient satisfaction. *World Neurosurg.* **2014**, *81*, 584–593.
18. Raouf, A.; Zidan, I. Endoscopic Removal of Third Ventricular Colloid Cyst: Experience of 90 Cases. *Neurosurgery Quarterly.* **2015**, *25(1)*, p 46–50. https://doi.org/10.1097/WNQ.0b013e3182a2fe78.
19. Sharifi, G.; Bakhtevari, M.H.; Samadian, M.; Alavi, E.; Rezaei, O. Endoscopic Surgery in Nonhydrocephalous Third Ventricular Colloid Cysts: A Feasibility Study. *World Neurosurg.* **2015**, *84*, 398–404.
20. Stachura, K.; Libionka, W.; Moskała, M.; Krupa, M.; Polak, J. Colloid cysts of the third ventricle. Endoscopic and open microsurgical management. *Neurol Neurochir Pol.* **2009**, *43(3)*, 251–7. PMID: 19618308.
21. Birski, M.; Birska, J.; Paczkowski, D.; Furtak, J.; Rusinek, M.; Rudas, M.; Harat, M. Combination of Neuroendoscopic and Stereotactic Procedures for Total Resection of Colloid Cysts with Favorable Neurological and Cognitive Outcomes. *World Neurosurg.* **2016**, *85*, 205–214.
22. Samadian, M.; Ebrahimzadeh, K.; Maloumeh, E.N.; Jafari, A.; Sharifi, G.; Shiravand, S.; Digaleh, H.; Rezaei, O. Colloid Cyst of the Third Ventricle: Long-Term Results of Endoscopic Management in a Series of 112 Cases. *World Neurosurg.* **2018**, *111*, e440–e448.
23. Brunori, A.; de Falco, R.; Delitala, A.; Schaller, K.; Schonauer, C. Tailoring Endoscopic Approach to Colloid Cysts of the Third Ventricle: A Multicenter Experience. *World Neurosurg.* **2018**, *117*, e457–e464.
24. Azab, W.A.; Abdelnabi, E.A.; Mostafa, K.H. Efficacy and Safety of the Rotational Technique for Endoscopic Transforaminal Excision of Colloid Cysts of the Third Ventricle. *World Neurosurg.* **2019**, *125*, e602–e611.
25. Vorbau, C.; Baldauf, J.; Oertel, J.; Gaab, M.R.; Schroeder, H.W.S. Long-Term Results After Endoscopic Resection of Colloid Cysts. *World Neurosurg.* **2019**, *122*, e176–e185.
26. Lin, M.; Bakhsheshian, J.; Strickland, B.; Rennert, R.C.; Chen, J.W.; Van Gompel, J.J.; Young, I.I.R.L.; Kumar, P.P.; Coppens, J.; Curry, W.T.; et al. Navigable Channel-Based Trans-Sulcal Resection of Third Ventricular Colloid Cysts: A Multicenter Retrospective Case Series and Review of the Literature. *World Neurosurg.* **2020**, *133*, e702–e710.
27. Stachura, K.; Grzywna, E.; Krzyżewski, R.M.; Kwinta, B.M. Retrospective evaluation of endoscopic treatment in colloid cyst of the third ventricle. *Videosurgery Other Miniinvasive Tech.* **2021**, *16*, 604–611.
28. Zymberg, S.T.; Riechelmann, G.S.; da Costa, M.D.S.; Ramalho, C.O.; Cavalheiro, S. Third ventricle colloid cysts: An endoscopic case series emphasizing technical variations. *Surg. Neurol. Int.* **2021**, *12*, 376.
29. Alkhaibary, A.; Baydhi, L.; Alharbi, A.; Alshaikh, A.A.; Khairy, S.; Abbas, M.; Aboushady, A.M.; Almuntashri, M.; Alassiri, A.H.; Alkhani, A.; et al. Endoscopic versus Open Microsurgical Excision of Colloid Cysts: A Comparative Analysis and State-of-the-Art Review of Neurosurgical Techniques. *World Neurosurg.* **2021**, *149*, e298–e308. https://doi.org/10.1016/j.wneu.2021.02.032.
30. Arnaout, M.M.; Elsamman, A.K. Perspectives on Endoscopic Transseptal Interforniceal Approach for Retroforaminal Colloid Cysts. *World Neurosurg.* **2021**, *152*, e71–e80.
31. Beaumont, T.L.; Limbrick, D.D.; Patel, B.; Chicoine, M.R.; Rich, K.M.; Dacey, R.G. Surgical management of colloid cysts of the third ventricle: A single-institution comparison of endoscopic and microsurgical resection. *J. Neurosurg.* **2022**, *137*, 905–913.

32. Roth, J.; Perekopaiko, Y.; Kozyrev, D.A.; Constantini, S.; Pediatric Colloid Cyst Study Group (PCCSG). Pediatric colloid cysts: A multinational, multicenter study. An IFNE-ISPN-ESPN collaboration. *J. Neurosurg. Pediatr.* **2022**, *29*, 543–550.
33. Peron, S.; Galante, N.; Creatura, D.; Sicuri, G.M.; Stefini, R. Use of a neuro-evacuation device for the endoscopic removal of third ventricle colloid cysts. *Front. Surg.* **2023**, *10*, 1214290.
34. Unal, T.C.; Sencer, A.; Dolas, I.; Gulsever, C.I.; Sahin, D.; Dolen, D.; Ozata, M.S.; Ozturk, M.; Aras, Y.; Aydoseli, A. Full-endoscopic removal of third ventricular colloid cysts: Technique, results, and limitations. *Front. Surg.* **2023**, *10*, 1174144.
